# Practical Laboratory Tools for Monitoring of BCR-ABL1 Transcripts and Tyrosine Kinase (TK) Domain Mutations in Chronic Myeloid Leukemia Patients Undergoing TK Inhibitor Therapy: A Single-Center Experience in Thailand

**DOI:** 10.31557/APJCP.2020.21.7.2003

**Published:** 2020-07

**Authors:** Nittaya Limsuwanachot, Adcharee Kongruang, Budsaba Rerkamnuaychoke, Roongrudee Singdong, Pimjai Niparuck, Saengsuree Jootar, Teerapong Siriboonpiputtana

**Affiliations:** 1 *Human Genetic Laboratory, Department of Pathology, Faculty of Medicine Ramathibodi Hospital, Mahidol University, Bangkok, Thailand. *; 2 *Doctoral Program in Clinical Pathology,* *Department of Pathology, Faculty of Medicine Ramathibodi Hospital, Mahidol University, Bangkok, Thailand. *; 3 *Department of Medicine, Faculty of Medicine Ramathibodi Hospital, Mahidol University, Bangkok, Thailand.*

**Keywords:** Chronic myeloid leukemia, BCR-ABL1 mRNA, BCR, ABL1 TKD mutations

## Abstract

**Objective:**

The genetic hallmark of CML is known as the appearance of t(9;22)(q34.1;q11.2) (BCR-ABL1) which is present in more than 95% of cases. Here, we demonstrated practical laboratory tools for monitoring of BCR-ABL1 transcripts in chronic myeloid leukemia patients undergoing TK inhibitor therapy.

**Methods:**

Real time quantitative PCR and direct sequencing were performed for monitoring of BCR-ABL1 transcripts in 245 treated CML.

**Results:**

At month 3 after first time point of monitoring, we found that 89% (218/245), 2% (5/245), and 9% (22/245) of patients are determined as optimal, warning, and failure response, respectively. The responses to TKI were slightly decreased at months 6 as following 73% optimal (180/245), 18% warning (43/245), and 9% failure response (22/245). Additionally, responses to TKI were gradually decreased at month 12 after first time point of monitoring as following 65% optimal (160/245), 13% warning (31/245), and 22% failure (54/245). We could detect 20% (49/245) of patients positive for *BCR-ABL1 *TKD mutations. Interestingly, one third (17 of 49) of TKD mutated cases were positive for compound/polyclonal mutation patterns. While major molecular response were observed in the majority of patients without TKD mutation, resistant to TKI were detected in patients with *T315I* mutation (n = 9; % mean IS = 8.1510, % median IS = 9.7000), compound/polyclonal mutations with T315I (n = 9; % mean IS = 13.0779, % median IS = 5.404), and other TKD mutations (n = 14; % mean IS = 8.1416, % median IS = 1.060), respectively.

**Conlusion:**

These practical laboratory techniques provided a more comprehensive understanding of CML progression during drug therapy and could be of benefit in earlier prognosis.

## Introduction

Chronic myeloid leukaemia is a clonal haematopoietic stem cells disorder characterized by the proliferation of malignant myeloid cells at all stages of differentiation. The genetic hallmark of CML is known as the appearance of the Philadelphia chromosome (Ph chromosome); t(9;22)(q34.1;q11.2) which is present in more than 90% of cases. The Ph chromosome is associated with the production of BCR-ABL1 chimeric protein with high ABL tyrosine kinase activity and recognized as a pathogenesis of the disease (Jabbour et al., 2008; Thompson et al., 2015). Patients with CML frequently have three phases of disease progression including the initial chronic phase, the transitional accelerated phase, and the progressive blast crisis phase (Calabretta and Perrotti, 2004; Tantiworawit et al., 2016). Since the discovery of imatinib (IM) (Baccarani et al., 2013; Buchdunger et al., 2001; Druker and Lydon, 2000), the drug has been shown to be the highly effective therapy in Ph positive CML. IM specifically blocks the ATP-binding site of BCR-ABL1 protein in leukemic cells resulting in inhibiting downstream signaling pathways. The first application of IM was to cure CML patients who were resistant to interferon-alpha (IFNα) (Goldman and Melo, 2003; Savage and Antman, 2002). The drug can induce a rapid haematological response and a major cytogenetic response in a chronic phase CML patients (Druker et al., 2001; Lim et al., 2017). In addition, IM could rise the overall survival and the event-free survival in the accelerated phase CML (Talpaz et al., 2002). Moreover, adverse effects of IM are generally mild when compared with interferon-alpha. IM could dramatically improve the overall survival of CML patient from about 20-80% to about 80-90%. Presently, treated CML patients are expected to live longer as well as have very well quality of life. Therefore, monitoring of the persistence specific surrogate disease biomarkers in CML (Ph chromosome, *BCR-ABL1* fusion gene, and BCR-ABL1 mRNA) is very important during a treatment to prevent disease relapse as well as to establish the end-point of therapy (Jabbour and Kantarjian, 2014). Recently, the European LeukemiaNet recommendations for the management of chronic myeloid leukemia suggested that bone marrow studies including cytogenetic analysis should be performed at 3, 6, and 12 months after starting therapy (Baccarani et al., 2013). Additionally, the improvement of clinical outcomes in treated CML patient are strongly associated with the deep and rapid molecular response. Hence, highly specific and sensitive techniques such as RT-PCR and real-time quantitative PCR (RQ-PCR) are important for routine monitoring of minimal residual disease (MRD) in treated patients (Breccia et al., 2016). Although IM has been proved for the effective treatment of CML, approximately to 20-30% of treated patients become IM resistance (Yang and Fu, 2015). Those patients become predominantly increasing in the BCR-ABL1 level. The resistance to IM treatment in CML patients could be categorized into two main mechanisms. Firstly, a *BCR-ABL1* dependent mechanism which is characterized by aberrations on the *BCR-ABL1* fusion gene, including *BCR-ABL1* kinase domain mutations (the most common cause of IM resistance), *BCR-ABL1* amplification, and clonal evolution. Secondly, a *BCR-ABL1* independent mechanism, which includes the impairment in function of specific IM transporters (influx and efflux transporters), drug concentration, the dysregulation of alternative signaling pathways, and epigenetics (An et al., 2010; Bixby and Talpaz, 2009; Chhikara et al., 2017; Cooper et al., 2009; Elias et al., 2013; Patel et al., 2017; Shah et al., 2016; Tauchi and Ohyashiki, 2004). At present, it is clear that the mutation in *ABL1* tyrosine kinase domain (TKD) is the major cause of IM resistance (Kaleem et al., 2015; Vacarean-Trandafir et al., 2019). Common TKD mutations are including the highly resistant mutations (Y253F/H, E255K/V, T315I, or H396P/R) which are recommended to alternative treatment options, the mildly resistant mutations (M244V, M351T, and F359V) which permit the improvement of clinical outcomes by increasing the IM dose to 600 or 800 mg/day (Hehlmann et al., 2007). Thus, molecular methods that can be able to early detect BCR-ABL1 kinase domain mutations are very important for monitoring of CML patients after treatment with tyrosine kinase inhibitors (TKIs). In this report, we described our routine RQ-PCR for the measurement of BCR-ABL1 mRNA and PCR sequencing technique for the detection of BCR-ABL1 TKD mutations in CML patients during TKIs treatments. Finally, we reported the frequency of *BCR-ABL1 *TKD mutations and the impact of clinical important TKD mutations on molecular response to TKIs treatment in Thai patients with CML.

## Materials and Methods

*Patients and samples*

This study was a retrospective analysis on a total of 245 CML patients during treatment at the Faculty of Medicine, Ramathibodi Hospital, Mahidol University, Bangkok, Thailand during January 2011 to December 2016. Cytogenetic study, RQ-PCR, and BCR-ABL1 kinase domain mutations screening were performed by the request from hematologists. The management of CML were based on the recommendations of European LeukemiaNet (Baccarani et al., 2009; Baccarani et al., 2006). Bone marrow and peripheral blood specimens were collected from patients at the time of diagnosis as well as during treatment. Standard karyotyping was mainly performed using bone marrow samples. The quantification of BCR-ABL1 mRNA levels were conducted by following the EAC protocol (Gabert et al., 2003). *BCR-ABL1 *kinase domain mutations analysis by PCR sequencing was performed on cDNA generated from RNA isolated from both peripheral blood and bone marrow samples. This work was approved by the ethic committee on human right related to research involving human subjects, Faculty of Medicine Ramathibodi Hospital, Mahidol University, Thailand and followed the principles of the Declaration of Helsinki (ID; MURA2020/174).

*Real-time quantitative PCR*

TaqMan-based real-time quantitative PCR (RQ-PCR) was performed according to a Europe Against Cancer Program (EAC) protocol to measure the BCR-ABL1 mRNA level during treatment (Gabert et al., 2003). Briefly, total RNA was isolated from peripheral blood/bone marrow samples of each patient by using TRIzol® Reagent (Thermo Fisher Scientific, USA) according to the manufacturer’s instructions and subsequently measured the concentration by using the Nanodrop 2000 spectrophotometer (Thermo Scientific, USA). cDNA was generated from a total of 1 μg RNA using SuperScript™ VILO™ cDNA Synthesis Kit (Thermo Fisher Scientific, USA) with random oligonucleotide primers. Real time quantitative PCR was performed on ABI 7500 Fast Real-Time PCR System (Thermo Fisher Scientific, USA). ABL1 was used as a house-keeping gene. BCR-ABL1/ABL1 copy ratio and % IS (international scale) of individual patient during treatment (every 3 months) were reported. The major molecular response (MMR) was defined as ≥ 3-log reduction (% IS ≤ 0.01) in BCR-ABL1/ABL1 ratio. The laboratory has achieved ISO15189 and 15190 accreditations and participated in the College of American Pathology (CAP) External Quality Assurance (EQA)/Proficiency Testing program. 

*The definition of response to TKIs*

The definition of response to any TKIs was followed the ENL 2013 recommendation guideline for the management of CML (Baccarani et al., 2013). The response to TKIs of individual patient could be assessed by both cytogenetic and molecular (RQ-PCR) techniques. The specific cut-off values at diverse time points of monitoring were clearly written in the guideline. The optimal response was associated with the achievement of finest long-term outcomes. Therefore, in this case, it was not necessary to change the treatment. In contrast, failure response meant that patient should change the treatment strategies to avoid the risk of disease progression and death. The middle between optimal and failure responses is called warning (previous known as sub-optimal response). In this group, patients required more frequent monitoring to allow appropriate changes for new treatments in a case of failure response. The guideline was able to be used for the definition of response to TKIs in all phases of CML (chronic, accelerated, and blast phases) and could be applied for the definition of response in second-line TKIs treatments. 

*BCR-ABL1 kinase domain mutation analysis*

PCR sequencing analysis for the detection of *BCR-ABL1* kinase domain mutations was adapted from the previous published protocol by Branford and Hughes, (2006) and Gabert et al., (2003). Briefly, semi-nested RT-PCR was performed to initially amplify BCR-ABL1 fusion transcripts (ENF501 and ABLR1) and subsequently amplify the* ABL1* kinase domain in a second round of PCR (ABLF1 and ABLR1). The PCR primers used in both semi-nested PCR and sequencing reaction were list in Table 1. The optimal PCR condition for both first and second rounds of semi-nested PCR was following the initial PCR with 95^o^C for 10 minutes, 40 cycles of 94^o^C for 1 minute, 68^o^C for 1 minute, 72^o^C for 2 minutes, and final extension at 72^o^C for 10 minutes. 5 µl of the first round PCR product was used as a template in the second round PCR. PCR product sizes of 1,464 or 1,539 base pairs for the first round PCR and 863 base pairs for the second round PCR were determined by 2 % agarose gel electrophoresis. PCR product (863 bp) was subsequently purified by using ExoSAP-IT™ PCR Product Cleanup Reagent (Thermo Fisher Scientific, USA) prior to cycle sequencing process. Sequencing reaction was performed by using BigDye^®^ Terminator v1.1 Cycle Sequencing Kit (Thermo Fisher Scientific, USA) and 1.6 pmol of each sequencing primer (ABLF1 and ABLR1). The amplified condition was following 96^o^C for 1 minute, 25 cycles of 96^o^C for 10 second, 50^o^C for 5 seconds, 60^o^C for 4 minutes. Sequencing product was purified using DyeEx 2.0 Spin Kit (QIAGEN, Germany) and subsequently sequenced with ABI 3130 Genetic Analyzer (Applied Biosystems, USA) according to the instruction protocol. Finally, sequence was compared to the wild-type *ABL *sequence (GenBank accession number X16416.1). 

*Statistical analysis*

Statistical analysis was performed using SPSS version 24.0 software (SPSS Inc., Chicago, IL, USA). Mean and median percent IS values of patients at 12 months of monitoring were analysed using independent T-test and Mann-Whitney test respectively. Statistical significance is accepted at p-values <0.05.

## Results

*The overall molecular response of 245 CML patients to TKI treatment*

**Figure 1:**
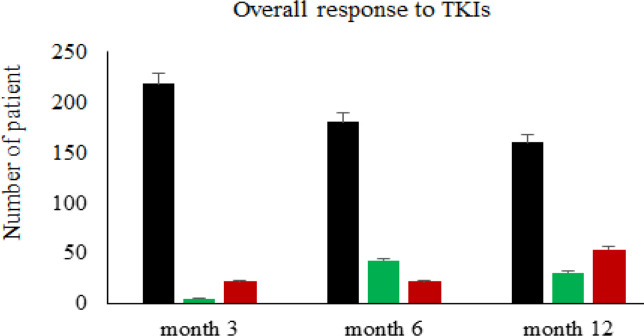
Clinical Response Categories of Chronic Myeloid Leukemia Patients (n = 245) to a 12-Month Tyrosine Kinase Inhibitor (TKI) Treatment at Ramathibodi Hospital, Mahidol University, Bangkok, Thailand. Definition of clinical response to TKI treatment was based on ENL 2013 recommendation guideline for management of CML (Baccarani et al., 2013). The colored-bar boxes indicate number of patient’s response to TKIs at specific time points of monitoring. The black boxes were patients with optimal response, green boxes were warning groups, and red boxes were patients with failure response to TKIs, respectively

We performed both cytogenetic analysis and real-time quantitative PCR on sequential samples collected from different time points of individual patients during treatment. The definition of response to tyrosine kinase inhibitors as a first-line treatment was based on the European LeukemiaNet recommendations for the management of chronic myeloid leukemia; 2013 (Baccarani et al., 2013). At month 3 of monitoring, we found that 89% (218/245), 2% (5/245), and 9% (22/245) of patients were determined as optimal, warning, and failure response, respectively. The overall responses to TKI treatment were slightly decreased at the month 6 of disease monitoring as following 73% of patients with optimal response (180/245), 18% of patients with warning (43/245), and 9% of patients with failure response (22/245). Additionally, we observed that the overall responses to TKI treatment were gradually decreased at month 12 of monitoring as following 65% of patients with optimal response (160/245), 13% of patients with warning (31/245), and 22% of patients with failure response (54/245) (Figure 1). *BCR-ABL1 *TKD mutations analysis was subsequently confirmed in cDNA samples of patients who exhibited signs of treatment failure. Using quantitative RT-PCR, we could determine the molecular response of CML patients after TKI treatment. 

*Mutation spanning of BCR-ABL1 TKD in treated CML patients*

**Figure 2:**
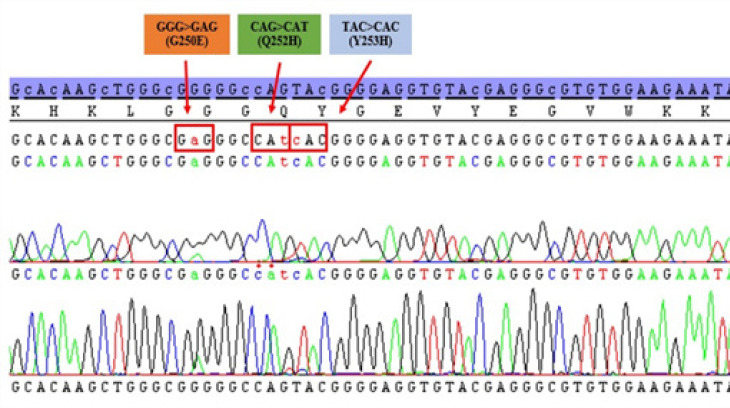
Electropherogram of *BCR-ABL1 *Tyrosine Kinase Domain Sequencing Profile from Patient with *G250E/Q252H/Y253H *Compound/Polyclonal Mutation

*BCR-ABL1* TKD mutations analysis by Sanger sequencing was performed in cDNA samples of patients with TKIs treatment failure. We could detect 20 % (49/245) of patients with positive results for *BCR-ABL1* TKD mutations. Interestingly, we found that one third (17 of 49) of TKD mutated cases were positive for compound/polyclonal mutation patterns. An example of electropherogram from patient with compound/polyclonal mutation was presented in Figure 2. The most frequent point mutation of *BCR-ABL1* TKD was the clinical relevant, T315I which was positive in 57% (28/49) of all mutated cases. Interestingly, compound/polyclonal mutations of *T315I *with other *BCR-ABL1 *TKD mutations were observed in nearly half of T315I mutated cases (12 of 28, 43%). Other point mutation types observed in this study were following 20% of E255K/V (10/49; 4 single and 6 compound/polyclonal mutations), 10% of F359V/I (5/49; 2 single and 3 compound/polyclonal mutations), 8% of F317L/I (4/49; 3 single and 1 compound/polyclonal mutations), 6% of Y253H (3/49; 1 single and 2 compound/polyclonal mutations), 4% of D276G (2/49; 1 single and 1 compound/polyclonal mutations), 4% of E459V/K (2/49), 2% of Q252H (1/49), 2% of F311I (1/49), 2% of A350T (1/49), 2% of E455K (1/49), 2% of F486S (1/49), respectively. Interestingly, *G250E* mutation was restricted positive as compound/polyclonal mutation patterns. There was no single mutation of *G250E* mutation observed in this study (Table 2).

*BCR-ABL1 TKD mutations and molecular response to TKI treatment*

**Figure 3:**
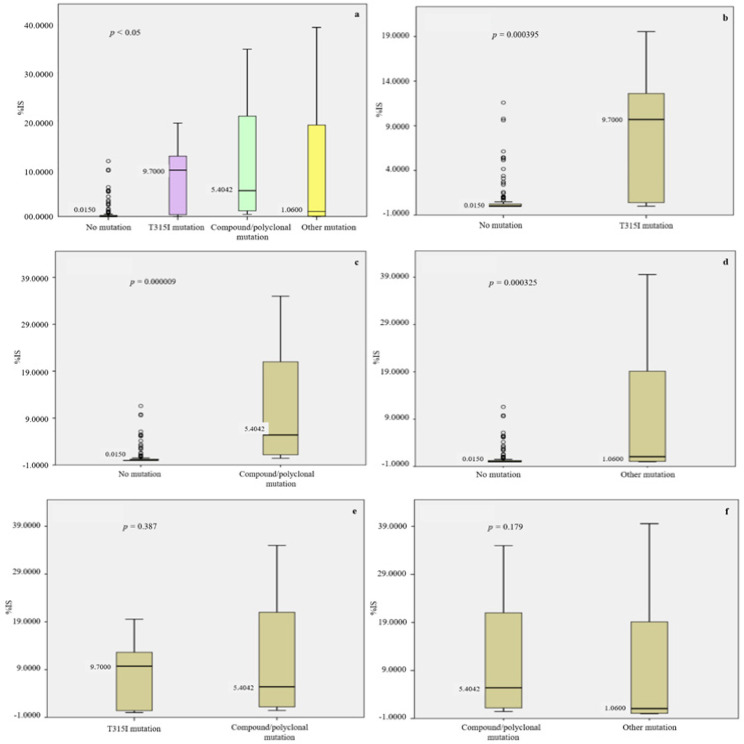
Statistical Analysis of *BCR-ABL1 *TKD Mutation Statuses and Molecular Response (median %IS) to Tyrosine Kinase Inhibitors at 12 Months of Monitoring. The overall statistical different in median %IS among groups as indicated *BCR-ABL TKD* mutation statuses (a). Comparison of median %IS between no *BCR-ABL1* TKD mutation and *T315I* (b). Comparison of median %IS between no *BCR-ABL1* TKD mutation and compound/polyclonal with *T315I* (c). Comparison of median %IS between no *BCR-ABL1 *TKD mutation and other *BCR-ABL1* *TKD* mutations (d). Comparison of median %IS between *T315I* mutation and compound/polyclonal with T315I (e). Comparison of median %IS between compound/polyclonal with *T315I* and other *BCR-ABL1* TKD mutations (f).

We further investigated the impact of *BCR-ABL1 *TKD mutations on the overall molecular response (by means of IS scale) to TKIs at month 12 of monitoring. 142 patients without *BCR-ABL1* TKD mutation were analysed. We observed that the median % IS of patients at month 12 is 0.0150 (< -4 log). This data suggested the majority of patients was achieved major molecular response after TKI treatment. However, there were few patients with a sign of molecular resistance at one year after treatment without *BCR-ABL1* TKD mutation. Because of *T315I* is well-recognized as a clinical significant point mutation that contributes to imatinib resistant in CML and it was predominantly observed in this study (57% of all mutated cases), we classified the treated patients into 4 main groups including patients without *BCR-ABL1* TKD mutation, *T315I* mutation, compound/polyclonal mutations with *T315I*, and other *TKD* mutations (such as *E255K, F317L, F359V/I*, and *F486S*). While major molecular response was observed in the majority of patients without *BCR-ABL1* TKD mutation, resistance to TKI treatments was detected in patients with *T315I* mutation (n = 9; % mean IS = 8.1510, % median IS = 9.7000), compound mutations with T315I (n = 9; % mean IS = 13.0779, % median IS = 5.404), and other *TKD* mutations (n = 14; % mean IS = 8.1416, % median IS = 1.060), respectively. Additionally, an increase of % IS to upper than 10 (> -1 log) was observed in nearly half of patients (44.44%) with both *T315I* single mutation and compound/polyclonal *T315I* mutations at 12 months of monitoring. Moreover, statistical analysis indicated that significant different in molecular response was observed in patients without *BCR-ABL1* TKD mutation compared with patients who carried *BCR-ABL1 *TKD mutations with T315I (p = 0.000395), compound/polyconal with T315I (p = 0.000009), and other type of *BCR-ABL1* mutations (p = 0.000325), respectively. However, it was not statistical different among patients with T315I, compound/polyconal with T315I, and other *BCR-ABL1* mutations observed in this study (table 3 and figure 3). Together, we could monitored the molecular response of CML patients after TKI treatment by serially measure the level of BCR-ABL1 fusion transcript and T315I mutation was predominantly identified in most cases with TKI resistant. 

**Table 1:** Primers for Semi-Nested RT-PCR and Direct Sequencing of *BCR-ABL1* TKD

Name	Sequence (5’-3’)	PCR process
ENF501	TCCGCTGACCATCAAYAAGGA	1^st ^round
ABLR1	TCCACTTCGTCTGAGATACTGGATT	1^st^ round, 2nd and sequencing
ABLF1	CGCAACAAGCCCACTGTCT	2^nd^ round, and sequencing

**Table 2:** Compound/polyclonal Mutations of *BCR-ABL1 TKD* Identified in This Study

Sample	*BCR-ABL1 TKD* mutation	L248	G250	Y253	E255	D276	V299	F311	T315	F317	F359	L384	H396	E459
AM01	L248V/del 248-274/T315I	L248V/del 248-274							T315I					
AM02	G250E/Y253H		G250E	Y253H										
AM03	G250E/Y253H/D276G		G250E	Y253H		D276G								
AM04	G250E/T315I		G250E						T315I					
AM05	G250E/E255K/T315I		G250E		E255K				T315I					
AM06	G250E/E255K/E255V/T315I		G250E		E255K/E255V				T315I					
AM07	Y253H/T315I			Y253H					T315I					
AM08	E255K/T315I				E255K				T315I					
AM09	E255V/T315I				E255V				T315I					
AM10	E255K/T317L				E255K					T317L				
AM11	E255V/F359I				E255V						F359I			
AM12	V299L/T315I/T317L						V299L		T315I	T317L				
AM13	V299L/L384M						V299L					L384M		
AM14	T315I/E459V								T315I					E459V
AM15	T315I/F359V								T315I		F359V			
AM16	T315I/F359V								T315I		F359V			
AM17	T315I/H396R								T315I				H396R	

**Table 3:** Statistical Analysis by Using Mean and Median %IS from RQ-PCR Data of Different Group of Patients as Indicated *BCR-ABL1* TKD Mutation Sstatuses at 12 Months of Monitoring

	*P*-value	
Compare variable type	Compare Mean (Independent T-test)	Compare Median (Mann-Whitney)
No Mutation & *T315I* Mutation	0.015	<0.05 (0.000395)
No Mutation & Compound/polyclonal Mutation with *T315I*	0.028	<0.05 (0.000009)
No Mutation & Others Mutation	0.05	<0.05 (0.000325)
*T315I* Mutation & Compound/polyclonal Mutation with *T315I*	0.364	0.387
*T315I* Mutation & Others Mutation	0.998	0.643
Compound/polyclonal Mutation with *T315I* & Others Mutation	0.393	0.179

**Table 4:** The Observed Frequency of *BCR-ABL1* TKD Mutations in Different Studies

Study	Method of the detection	Overall frequency of *BCR-ABL1* mutations (%)	Common hotspot *BCR-ABL1* TKD mutations (%)	CML phases
This study (Thai study)	Direct sequencing	20 (49/245)	Single mutation (13) Q252H (0.4) F317L (1) Y253H (0.4) A350T (0.4) E255K (2) F359I/V (1) D276G (0.4) E455K (0.4) F311I (0.4) E459K (0.4) T315I (7) F486S (0.4) Compound/polyclonal mutations (7)	NA
Elias et al., (2014 )(Malaysian study) (Elias et al., 2014)	D-HPLC and Direct sequencing	22 (28/125)	Single mutation G250E (2) M351T (2) G251E (1) E355A/G (2) Y253H (1) F359C (2) E255K (4) N368S (1) D276G (1 L387M (1) V289F (1) H396R (1) T315I (7) A397P (1) Compound/polyclonal mutation (NA)	CP,AP,BP
Meggyesi et al., (2012)	Direct sequencing	20 (15/74)	Single mutation	CP,AP,BP, Ph positive ALL
(Hungarian study) (Meggyesi et al., 2012)			M244K/V (8) M351T (5) G250E (1) E355G (1) Y253H (1) F359I/V (3) E255V (4) L384M (1) D276G (1) L387M (1) E279K (1) H396R (1) T315I (5) Compound/polyclonal mutation (NA)	
Kim et al., (2009) (Korean syudy) (Kim et al., 2009)	Direct sequencing and ASO-PCR	51 (70/137)	Single mutation M244V (9) E355G (1) G250E (10) F359C (1) Q252H (4) F359I/V (5) Y253F/H (14) H396P/R (2) E255K/V (17) S417Y (1) T315I (23) E450K (1) F317L (4) E459K (4) M351T (2) P480L (1) Compound/polyclonal mutation (NA)	CP,AP,BP
Branford et al., (2003) (Australian study) (Branford et al., 2003)	Direct sequencing	19 (27/144)	Single mutation (14) M244V (1) M351T (6) L248V (1) E355G (2) G250E (1) F359V (1) Q252H (3) H396R (1) Y253F (1) S417Y (1) E255K/V (4) E459K (1) T315I (1) F486S (1) F317L (1) Compound/polyclonal mutations (5)	CP,AP

## Discussion

During the past two decades, treatment of BCR-ABL1 positive CML has dramatically improved since the development of imatinib and other tyrosine kinase inhibitors. While the majority of patients remain in prolonged complete molecular response, nearly one third of patient develops an acquired *TKD* mutations resulting in disease relapse. Several laboratory techniques including conventional cytogenetic, FISH, RT-PCR, quantitative RT-PCR, and digital droplet PCR have been broadly used for routine monitoring of Philadelphia positive CML during the treatment. Furthermore, PCR sequencing and recently next generation sequencing technique have been subsequently performed to detect *BCR-ABL1* TKD mutational profile in patients who exhibit signs of TKI resistance. Since 2004, we had established a laboratory testing panel for a comprehensive monitoring of CML patients in the country which is continually improved according to the ELN guidelines. Those are including standard karyotyping, commercially dual-color FISH probes, and laboratory developed-test series of RT-PCR for BCR-ABL1, real-time quantitative PCR for BCR-ABL1 mRNA, and direct sequencing technique for *BCR-ABL1 *TKD mutations. As the quality of each laboratory test is very important, all of those assays have been accredited by the International Organization for Standardization (ISO15189 and ISO15190) as well as participated in the College of American Pathology (CAP) External Quality Assurance (EQA)/Proficiency Testing program. 

Perspective of cytogenetic analysis for the diagnosis and monitoring of CML is still limited used and routinely service in Thailand and nearby countries. We use the International System for Human Chromosome Nomenclature (ISCN2016) as a guideline for the description of individual CML karyotype. The main advantages of conventional cytogenetic analysis in CML are including; it is still a gold-standard method for the detection of Ph chromosome and it could detect several additional chromosomes in both newly diagnosed (baseline) and treated patients. Therefore, the assay is still mandatory for the monitoring of clonal evolution during treatment. However, the technique has some drawbacks such as low resolution, low sensitivity, time consumable, and laborious technique. The molecular cytogenetic, FISH is not frequently used in our laboratory for both diagnosis and monitoring of CML due to the assay is not provide much clinical relevant data compared with conventional cytogenetic analysis. However, we use FISH in special scenarios such as for the monitoring of Ph positive CML/AML with rare BCR-ABL1 transcriptional variants. 

Since the announcement of European Against Cancer (EAC) procedure for standardization and quality control of real-time quantitative PCR for minimal residual disease detection in leukemia (Gabert et al., 2003), we had followed this valuable protocol for the detection of several fusion transcripts including the BCR-ABL1 major (p210) transcript. This protocol have been proved to be practically used for monitoring CML patient during treatment because it has several benefits such as high specificity and sensitivity, easy to perform, economy, and short turn-around time. More importantly, the result from RQ-PCR is very informative and representing to disease progression/response in individual patient during the treatment. For the international standardization, we could achieve the conversion factor for international scale calculation in 2014 by the support from the Division of Genetics & Molecular Pathology, Royal Adelaide Hospital, Australia. Here, based-on the ELN 2013 guideline (Baccarani et al., 2013), we serially monitored (every 3 months) BCR-ABL1 mRNA by using RQ-PCR in 245 CML patients after treatment. We observed that the overall response to TKI treatment in patients were gradually decreased at the month 12 after first time point of monitoring (optimal response started from 89% at month 3 and turned out to be 65% at month 12). Moreover, we found that 22% of patients were categorized as failure to TKI at month 12 by monitoring with RQ-PCR. Those patients were successively analyzed the BCR-ABL1 TKD profiles using PCR sequencing. While we did not have much clinical therapeutic data of individual patients, we could perform the robust RQ-PCR method for effective monitoring of BCR-ABL1 mRNA in CML during treatment. 

Perspective of the detection of *BCR-ABL1* TKD mutations in treated patients, we found that 20% of patients are positive for *BCR-ABL1* TKD mutations by mean of using direct sequencing technique. Our data was similar to some of previous publications regarding to the overall frequency of *BCR-ABL1* TKD mutations in treated CML. Those were including in the Australian study (19%) (Branford et al., 2009), Argentinean study (23%) (Bengio et al., 2011), and Malaysian study (22.4%) (using dHPLC and direct sequencing) (Elias et al., 2014). However, there were some publications reported that the observed in frequency of *BCR-ABL1* TKD mutations are range from 30% to 65% (Cortes et al., 2007; Markose et al., 2009; Qin et al., 2011). The overall frequency of *BCR-ABL1 *TKD mutation in treated CML patients from various studies were summarised in table 4. The difference in distribution of *BCR-ABL1* TKD mutations from various studies could be explained by the variation in number and inclusion criteria of selected case, treatment protocol, phase of disease, and the method of used for the detection of *BCR-ABL1* TKD mutations. Similar to previous reports (Branford et al., 2009; Branford et al., 2003; Markose et al., 2009; Nicolini et al., 2006), the most frequents *BCR-ABL1* TKD mutations involving in the establishment of TKI resistance by mean of RQ-PCR was the T315I which was accounted for 57% of all mutated cases. Interestingly, we found that 35% of all mutated cases persisted compound/polyclonal mutation patterns with dominantly harbour co-mutation of *T315I*. Additionally, patients with T315I compound/polyclonal mutations showed higher %IS and might had phenotypic resistance than those with *T315I* alone and other *TKD* mutation patterns at month 12 of monitoring. Although we had no high performance technology such as digital droplet PCR and next generation sequencing to deeply and massively monitor *BCR-ABL1* TKD mutation profile, we could perform the effective laboratory developed test (direct sequencing) for the detection of *BCR-ABL1* TKD mutations in our tested samples. Based on this observation data, we are going to further investigate the impact of compound/polyclonal mutations in the treatment of *CML *in the context of precision medicine in recent future. 

In summary, we described the practical laboratory tools for routinely monitoring of CML patients during treatment. Serially measurement of BCR-ABL mRNA using RQ-PCR was able to effectively monitor the dynamic change of residual disease in treated patients. Moreover, we could identify several *BCR-ABL1* TKD mutations in patients who failed to TKI therapy. Patients with *T315I* compound/polyclonal mutations had high level of %IS than those with T315I alone, others *TKD *mutations, and without *TKD* mutation. Regarding to ELN 2013 guideline, the integration of laboratory tools including cytogenetic analysis, iFISH, RQ-PCR and direct sequencing is critical for the comprehensive monitoring of Ph positive CML during treatment. 
